# Psychological Distress Symptoms Associated With Life Events in Patients With Bipolar Disorder: A Cross-Sectional Study

**DOI:** 10.3389/fpsyt.2018.00200

**Published:** 2018-05-23

**Authors:** Aiko Sato, Tasuku Hashimoto, Atsushi Kimura, Tomihisa Niitsu, Masaomi Iyo

**Affiliations:** ^1^Department of Psychiatry, Chiba University Graduate School of Medicine, Chuo-ku, Japan; ^2^Department of Psychiatry, Sodegaura Satsukidai Hospital, Sodegaura, Japan

**Keywords:** bipolar disorder, depression, life events, mania, psychological distress

## Abstract

Stressful life events, although less serious than traumatic experiences, affect the clinical course of patients with bipolar disorder. We previously found that bipolarity in patients with major depression is related to the severity of psychological distress symptoms associated with onset-related events. Here, we investigated whether, and to what extent, bipolar patients perceive stressful events as psychological distress symptoms, specifically, intrusion, avoidance, and hyperarousal. Further, we investigated the relationship between the clinical features and the severity of psychological distress symptoms associated with stressful life events, according to mood states. We recruited 79 bipolar patients (depression group, *n* = 32; mania, *n* = 22; euthymia, *n* = 25) in this cross-sectional study. We adopted the Impact of Event Scale-Revised (IES-R) to assess the severity of psychological distress symptoms associated with past stressful events. We also evaluated the Hamilton Depression Rating Scale (HDRS) and the Young Mania Rating Scale (YMRS). The mean (standard deviation) IES-R scores of bipolar patients with a depressive episode (38.06 [16.56], *p* = 0.0005) and of those with a manic/hypomanic episode (44.56 [24.14], *p* = 0.004) were significantly higher than of those with euthymia (19.81 [12.86]). The HDRS, but not the YMRS, scores showed significant correlations with the IES-R scores, regardless of mood episodes (depression group, *r* = 0.42; mania, *r* = 0.64; euthymia, *r* = 0.70). This study demonstrates that bipolar patients with a manic/hypomanic or depressive episode perceive stressful life events as more severe psychological distress symptoms than do euthymic patients. Moreover, in patients with bipolar disorder, the severity of depressive symptoms, but not of manic symptoms, is positively correlated with that of the psychological distress symptoms, regardless of their mood episodes or euthymic state. Therefore, depressive symptoms may be closely related to the psychological distress symptoms associated with stressful past events in patients with bipolar disorder.

## Introduction

It is well-recognized that stressful life events affect vulnerability, onset, and relapse or recurrence of bipolar disorder (1–4). A previous study has reported that the prevalence of stressful life events in patients with bipolar disorder is higher than that in healthy people (5). Further, social problems, such as a protracted stressful life events, disturb symptomatic remission or recovery of patients with bipolar disorder (3). Although the stressful life events severely affect onset; clinical exacerbation, including relapse; and prognosis of bipolar disorder, only a few studies have investigated how the patients are distressed by them, and to what extent they experience psychological symptoms with intrusive or unpleasant memories of such events. Therefore, it is necessary to investigate clinical features associated with stressful life events in patients with bipolar disorder in the context of psychiatric symptomatology, to develop a better understanding and management strategy against bipolar disorder.

Stressful life events, which occur more frequently, and are less serious than traumatic experiences meeting the diagnostic criteria for posttraumatic stress disorder (PTSD), cause symptoms of intrusion, avoidance, and hyperarousal, which are similar to those observed in PTSD, in adults and adolescents (6, 7). Our previous study reported that patients with treatment-refractory or non-remitting depression suffered from psychological distress symptoms (PDSs), such as intrusion and avoidance, associated with onset-related life events, which alone did not lead to fatal outcomes, compared to PDS combined with remitted depression (8). This study also examined the association between the severity of PDSs that were associated with onset-related life events and depressed patients' bipolarity; bipolarity was defined as patients satisfying the criteria of either “bipolar spectrum disorder” (9) or “bipolar specifier” (10, 11), as described previously (8). Our previous findings indicated that patients with depression and bipolarity are more likely to suffer from PDSs associated with onset-related life events than those without bipolarity (8). Based on this knowledge, we hypothesized that patients with bipolar disorder experience PDSs associated with stressful life events as much as patients with treatment-resistant or non-remitted unipolar depression. Given that many patients with bipolar disorder, without comorbidity of PTSD or experience of traumatic events meeting the diagnostic criteria of PTSD, experience more stressful life events than healthy people (5); recognition of PDSs associated with such cases is important for understanding the pathophysiology of the clinical features of bipolar disorder.

The purpose of this study was to identify PDSs in patients with bipolar disorder. In the present study, we defined PDSs as consisting of intrusions, avoidance, and hyperarousal, associated with the past stressful events experienced by the patients that are more mundane and less serious than those satisfying the PTSD diagnostic criteria A in the fifth edition of the Diagnostic and Statistical Manual of Mental Disorders (DSM-5) (12), objectively. We also investigated the relationships between the clinical features of bipolar disorder and the severity of PDSs associated with stressful life events according to mood states (depression, mania or hypomania, and euthymia).

## Materials and methods

### Study design

This study employed a cross-sectional design.

### Ethics statement

This study was approved by the ethics committees of Chiba University Graduate School of Medicine, Kisarazu Hospital, and Sodegaura Satsukidai Hospital. All subjects provided written informed consent for their participation in this study after the protocol had been fully explained to them. All experiments were performed in accordance with the Helsinki Declaration.

### Participants and procedures

This study was conducted between July 2016 and July 2017. Participants were recruited from among patients commuting to or hospitalized in Chiba University Hospital, Kisarazu Hospital, or Sodegaura Satsukidai Hospital. The patients' ages ranged from 20 to 65 years, and they were diagnosed with bipolar disorder according to the DSM-5 criteria (12) using the Japanese version of the Mini International Neuropsychiatric Interview (13, 14). We excluded patients under 20 or over 65 years of age, patients with PTSD, schizophrenia, major depressive disorder, comorbid dementia, organic mental disorder, neurodevelopmental disorders, or impending suicide attempt.

We selected the target sample size of the present study based on our previous study (8). Consequently, a total of 210 outpatients and 17 inpatients underwent eligibility screening for this study. Of these, 118 patients did not meet the criteria for eligibility, and 109 patients were eligible to participate. Of these, 30 patients declined an interview. Finally, 79 patients were included in this study. We classified these patients into 3 groups, according to mood state: depression, mania or hypomania, and euthymia.

### Assessment of depression and mania

We assessed the severity of depression using the Structured Interview Guide for the 17-item Hamilton Depression Rating Scale (HDRS) (15, 16), and evaluated the severity of mania using the Young Mania Rating Scale (YMRS) (17). Euthymia was defined by an HDRS score ≤ 7 and a YMRS score ≤ 7. We categorized mixed state patients with HDRS and YMRS scores >7 into a depressive or manic state, according to the DSM-5.

### Assessment of clinical characteristics

We assessed demographic data, such as age, sex, comorbidity, physical disease, family history of psychiatric disorders in first-degree relatives, years of education, employment history, current employment, marital history, history of smoking, history of alcohol drinking, history of substance use, present medication, disease and therapy duration, type of bipolar disorder (bipolar I disorder or bipolar II disorder), and clinical features of the current or past episodes (with anxious distress, mixed features, rapid cycling, melancholic features, atypical features, psychotic features, catatonia, peripartum onset, and seasonal pattern), according to the DSM-5 definition. In the current study, physical diseases included patients under treatment for hypertension, diabetes, hyperlipidemia, reflux esophagitis, gastric ulcer, hypoferric anemia, asthma, gout, lumbar disc hernia, premenstrual syndrome, and sleep apnea syndrome.

### Assessment of stressful life events

#### Interview procedure for assessing stressful life events

We had already excluded patients with PTSD, as this was one of the study exclusion criteria. Moreover, we directly asked patients whether they suffered from PTSD according to the Japanese version of the Mini International Neuropsychiatric Interview and the Japanese version of the Structured Clinical Interview for DSM-IV (18).

After excluding patients with PTSD, as described above, we assessed whether patients experienced “life events related to PDSs” (here, referred to as “stressful life events”) by asking the following question, “Did you experience life events resulting in nightmares, flashbacks, involuntary and intrusive memories, or persistent effortful avoidance, excluding life events matching PTSD criteria?” We also asked whether patients experienced onset-related events; these events were regarded as general events that the patients themselves recognized as events that could trigger the onset, irrespective of the presence or absence of current PDSs.

#### Categorizing life events

We classified the patients' life events into 10 groups by referring to the list of threatening experiences in a questionnaire that is frequently used to assess stressful events (19) as follows: family problems without abuse, separation from a close person, interpersonal-related events, health-related events, money-related events, sex-related events, change of living conditions, job-related events, bullying or neglect, and other events.

#### Measure of the severity of PDSs associated with stressful life events

The impact of event scale-revised (IES-R) is a self-reported questionnaire for assessing the severity of psychological symptoms related to stressful life events (20). The IES-R has been developed to assess trauma-related symptoms in patients with PTSD. It consists of 22 items, including 8 for intrusion symptoms, 8 for avoidance, and 6 for hyperarousal, which are the 3 major sub-categories of PTSD symptoms. Each of the items is scored from a scale of 0–4, with the higher scores implying greater severity of traumatic symptoms. Therefore, the total score for the IES-R ranges from 0 to 88. The IES-R has been validated, with ensuring internal consistency worldwide (21), and the Japanese version has also been developed and is available (22).

As noted above, we hypothesized that patients with bipolar disorder could perceive PDSs associated with their life events in a manner similar to patients with PTSD and patients with unipolar depression, as reported in our previous study (8). Therefore, we adopted the IES-R to evaluate PDSs in this study. We instructed the patients to write their stressful life events into the blank space of the introductory document of the IES-R, and to answer each item of the IES-R regarding their life event, as described above. In addition, we also instructed the patients to write down their onset-related event, and to answer each item in terms of their onset-related event.

### Primary and secondary endpoints

The primary endpoint was the prevalence of subjects with PDSs associated with life events among the patients with bipolar disorder. The secondary endpoints were the comparison of the IES-R scores in each group, as classified by mood states, and the relationships between the IES-R scores and clinical features.

### Statistical analysis

We analyzed the data separately for the 3 groups (depression, mania or hypomania, and euthymia groups). We performed all analyses using SPSS for Windows, Version 19 (IBM Corp., Armonk, NY, USA). The chi-square or Fisher's exact test was used for categorical variables, and Student's *t*-test or one-way analysis of variance (one-way ANOVA) for the other variables. We performed one-way ANOVA for total and sub-category scores of the IES-R, HDRS, and YMRS, followed by Games Howell test for multiple comparisons. We also tested the correlation between IES-R and HDRS scores, and between IES-R and YMRS scores using Pearson's product moment correlation analysis. The level of significance was set at *p* < 0.05, and the power was set at 0.80.

## Results

### Patients' characteristics

Table 1 shows the characteristics of the participants included in the analysis. The 79 patients with bipolar disorder were categorized into 3 groups: the depression group (*n* = 32), mania or hypomania group (*n* = 22), and euthymia group (*n* = 25). There were no significant differences in age, sex, and years of education among the 3 groups. Further, there were no significant differences in employment history, current employment, marital history, physical diseases, and first-degree relatives with psychiatric disorders among the 3 groups. However, there were significant differences in the proportion of inpatients and outpatients, and psychiatric comorbidity. Table 2 shows the categorization of psychiatric comorbidities. In all groups, the most common comorbidity was panic disorder. Three patients in the depression and mania group exhibited 2 psychiatric comorbidities, and 1 patient in the depression group exhibited 3 psychiatric comorbidities. In the euthymia group, no patient exhibited more than 1 comorbidity. There were significant differences in the mixed and the melancholic features, and no significant differences in other clinical features (Table 1).

**Table 1:** Patient characteristics, based on patient groups (depression group, mania or hypomania group, and euthymia group).

	**Depression (*n* = 32)**	**Mania^a^ (*n* = 22)**	**Euthymia (*n* = 25)**	***p*-value**
Age, years (SD)	45.0(10.0)	43.4(12.2)	47.8(11.1)	NS
[Age range] (years)	[24-63]	[20-64]	[26-64]	
Sex, male/female	16/16	13/9	11/14	NS
Outpatient/In-patient	27/5	14/8	24/1	0.01^d^
Education, years (SD)	13.8(2.2)	13.2(2.6)	13.6(2.0)	NS
Employment history (%)	29(90.4)	21(95.5)	24(96.0)	NS
Current employment (%)	15(46.9)	7(31.8)	13(52.0)	NS
Marital history (%)	16(50.0)	8(36.4)	14(56.0)	NS
Smoking (%)	8(25.0)	9(40.9)	8(32.0)	NS
Alcohol intake (%)	8(25.0)	6(27.3)	5(20.0)	NS
Substance use (%)	0(0.0)	1(4.5)	2(8.0)	NS
Physical disease (%)	14(43.8)	13(59.1)	11(44.0)	NS
Psychiatric comorbidity (%)	21(65.6)	13(59.1)	5(20.0)	0.001^d^
Family psychiatric history^b^ (%)	9(28.1)	9(40.9)	9(36.0)	NS
Type, Bipolar I/II	11/21	13/9	10/15	NS
**Clinical features^c^**				
With anxious distress (%)	0(0.0)	0(0.0)	1(4.0)	NS
With mixed features (%)	2(6.3)	9(40.9)	3(12.0)	0.003^d^
With rapid cycling (%)	0(0.0)	0(0.0)	0(0.0)	NS
With melancholic features (%)	14(43.8)	3(13.6)	5(20.0)	0.03^d^
With atypical features (%)	2(6.3)	0(0.0)	0(0.0)	NS
With psychotic features (%)	8(25.0)	7(31.8)	9(36.0)	NS
With catatonia (%)	0(0.0)	0(0.0)	0(0.0)	NS
With peripartum onset (%)	0(0.0)	0(0.0)	1(4.0)	NS
With seasonal pattern (%)	2(6.3)	0(0.0)	0(0.0)	NS
Disease duration, years (SD)	16.0(7.9)	18.9(10.5)	16.2(10.9)	NS
Therapy duration, years (SD)	12.8(7.7)	12.7(8.6)	13.1(10.3)	NS
HDRS, points (SD)	14.6(4.9)	7.9(4.9)	3.2(2.0)	4.0 × 10^−15^^d^
YMRS, points (SD)	1.9(1.9)	13.7(5.0)	1.2(1.7)	1.0 × 10^−14^^d^

*Variables represent mean (standard deviation: SD)*.

*HDRS, Hamilton Depression Rating Scale; YMRS, Young Mania Rating Scale; NS, not significant*.

a *Including mania and hypomania patients*.

b *Family history of psychiatric disorder in a first-degree relative*.

c *Clinical features include current or past episode and may overlap*.

d *The data for the three groups were analyzed using one-way ANOVA, followed by the Games–Howell test for multiple comparisons*.

**Table 2:** Psychiatric comorbidities, based on patient groups.

	**Depression (*n* = 32)**	**Mania^a^ (*n* = 22)**	**Euthymia (*n* = 25)**	**All (*n* = 79)**
Psychiatric comorbidity (%)	21 (65.6)	13 (59.1)	5 (20.0)	39 (49.4)
Panic disorder (%)	9 (28.1)	5 (22.7)	2 (8.0)	16 (20.3)
Social anxiety disorder (%)	7 (21.9)	3 (13.6)	1 (4.0)	11 (14.0)
Obsessive-compulsive disorder (%)	4 (12.5)	2 (9.1)	0 (0)	6 (7.6)
Alcohol dependence (%)	2 (6.3)	3 (13.6)	1 (4.0)	6 (7.6)
Bulimia nervosa (%)	2 (6.3)	1 (4.5)	0 (0)	3 (3.8)
Anorexia nervosa (%)	0 (0)	1 (4.5)	0 (0)	1 (1.3)
2 psychiatric comorbidities (%)	3 (9.4)	3 (13.6)	0 (0)	6 (7.6)
3 psychiatric comorbidities (%)	1 (3.1)	0 (0)	0 (0)	1 (1.3)

a *Including mania and hypomania patients*.

Table 3 shows the prevalence of subjects experiencing life events. Fifty-six subjects (70.9% of all subjects) experienced both a stressful life event and an onset-related event. Eleven out of the 56 subjects answered that their stressful life event and onset-related event was the same event. A further 23 subjects (29.1%) experienced either a stressful life event or an onset-related event. All subjects experienced at least one of these events (Table 3).

**Table 3:** The prevalence of subjects with life events.

**Stressful life events**	**Onset-related events**	**Depression (*n* = 32)**	**Mania^a^ (*n* = 22)**	**Euthymia (*n* = 25)**	**All (*n* = 79)**
Yes	Yes	28	14	14	56
		[87.5%]	[63.6%]	[56.0%]	[70.9%]
Yes	No	3	2	2	7
		[9.4%]	[9.1%]	[8.0%]	[8.9%]
No	Yes	1	6	9	16
		[3.1%]	[27.3%]	[36.0%]	[20.3%]
No	No	0	0	0	0
		[0.0%]	[0.0%]	[0.0%]	[0.0%]

a *Including mania and hypomania patients*.

Table 4 shows the classification of life events for all patients. In terms of stressful life events, there were differences in the events most commonly experienced by patients among the 3 groups. Conversely, for onset-related events, the events most commonly experienced by patients were job-related events in the depression and euthymia group, while many patients experienced interpersonal-related events, changes in living conditions, or job-related events. For onset-related events, 30.6% of all patients reported experiencing overlapping events.

**Table 4:** Classification of life events.

	**Stressful life events**			**Onset-related events**		
	**Depression (*n* = 31)**	**Mania^a^ (*n* = 16)**	**Euthymia (*n* = 16)**	**Depression (*n* = 29)**	**Mania^a^ (*n* = 20)**	**Euthymia (*n* = 23)**
Family problems with no abuse	1	4	1	1	1	0
Separation from close person	3	4	5	3	2	1
Interpersonal-related events	7	3	3	8	4	8
Health-related events	8	0	4	4	2	5
Money-related events	1	0	0	2	0	0
Sex-related events	2	1	0	0	0	0
Change of living conditions	0	0	0	2	7	7
Job-related events	2	0	2	9	5	11
Bullying, neglect	6	3	0	4	4	0
Other events	2	1	1	1	2	1
Overlapping events	1	1	0	4	8	10

a *Including mania and hypomania patients*.

Table 5 shows the medication profiles for all participants. The predominant profile for all groups (34.4% in depression, 59.1% in mania or hypomania, and 48.0% in euthymia group) was a combination of mood stabilizers and antipsychotics. The highest proportion of patients treated with a combination of mood stabilizers, antipsychotics, and antidepressants was observed in the depression group (21.9%). Only 1 patient in the euthymia group was drug-free.

**Table 5:** Medication profiles of the three patient groups.

**Class of medication**	**Depression (*n* = 32)**	**Mania^a^ (*n* = 22)**	**Euthymia (*n* = 25)**	**All (n = 79)**
**MOOD STABILIZERS (MS)**				
Lithium	11	12	12	35
Sodium valproate	6	11	5	22
Lamotrigine	18	7	7	32
Topiramate	1	0	0	1
Gabapentin	0	1	0	1
Total (MS)	36	31	24	91
**ANTIPSYCHOTICS (AP)**				
Olanzapine	3	3	2	8
Quetiapine	8	6	5	19
Aripiprazole	10	6	8	24
Other	4	6	2	12
Total (AP)	25	21	17	63
**ANTIDEPRESSANTS (AD)**				
SSRI	8	3	1	12
SNRI	5	0	2	7
NaSSA	1	0	1	2
Trazodone	1	0	1	2
Other	1	0	0	1
Total (AD)	16	3	5	24
Benzodiazepine (BZ)	27	24	15	66
**MEDICATION COMBINATION**				
MS	6	4	5	15
AP	2	1	2	5
AD	0	0	0	0
MS + AP	11	13	12	36
MS + AD	3	1	2	6
AP + AD	3	1	1	5
MS + AP + AD	7	2	2	11
Drug-free	0	0	1	1

*MS, Mood stabilizers; AP, Antipsychotics; AD, Antidepressants; SSRI, Selective Serotonin Reuptake Inhibitor; SNRI, Serotonin and Noradrenaline Reuptake Inhibitor; NaSSA, Noradrenergic and Specific Serotonergic Antidepressant; BZ, Benzodiazepine*.

a *Including mania and hypomania patients*.

### IES-R scores for life events among the 3 groups

#### IES-R scores for stressful life events

**Figure 1:**
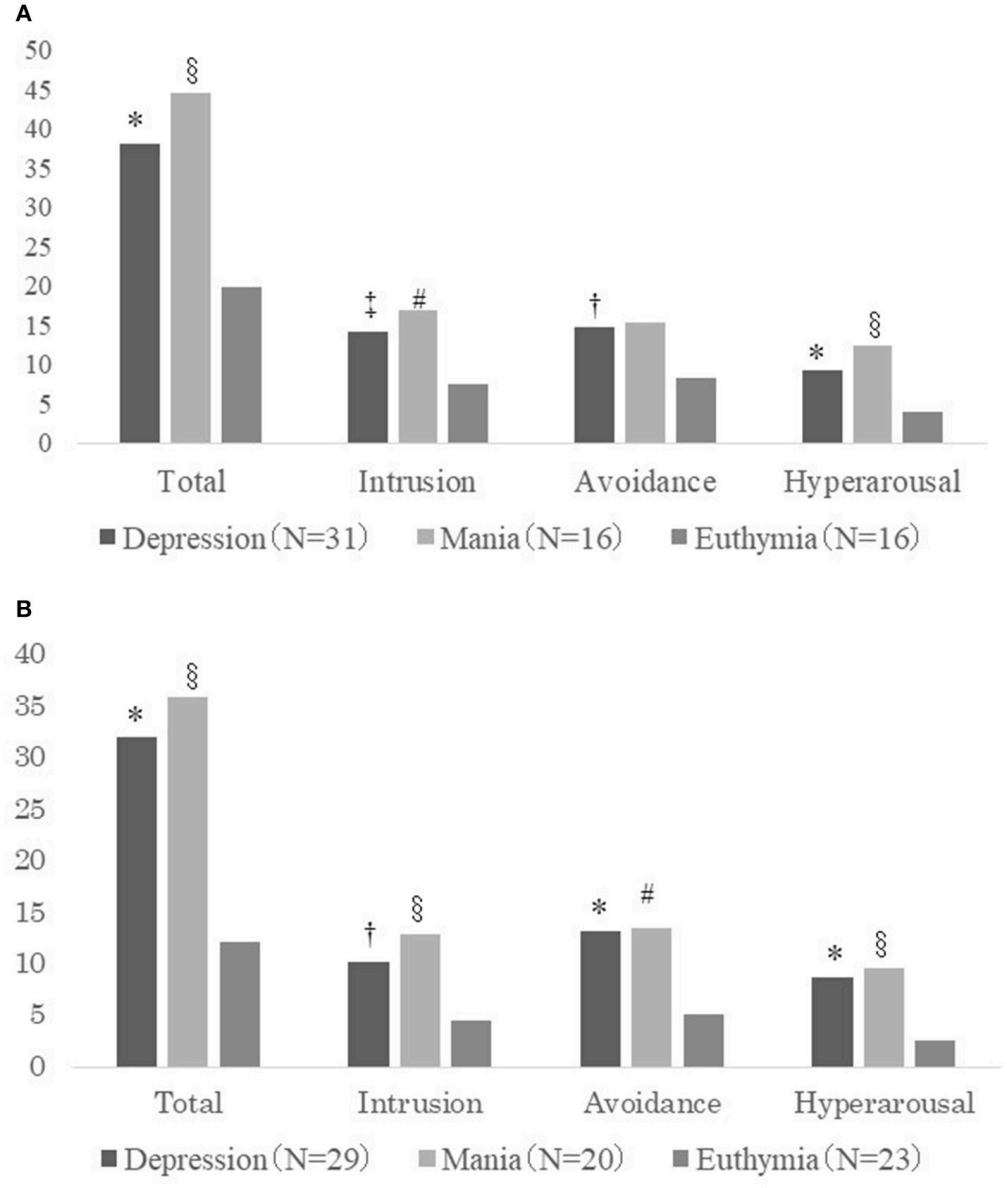
**(A)** IES-R scores for life events related to PDSs (total and sub-categories: intrusion, avoidance, and hyperarousal) for the depression, mania, and euthymia groups. ^*^Comparison between the depression and euthymia groups (*p* < 0.001); †comparison between the depression and euthymia groups (p < 0.01); ‡comparison between the depression and euthymia groups (*p* < 0.05); §comparison between the mania and euthymia groups (*p* < 0.01); #comparison between the mania and euthymia groups (*p* < 0.05). **(B)** IES-R scores for onset-related events (total and sub-categories: intrusion, avoidance, and hyperarousal) for the depression, mania, and euthymia groups. ^*^Comparison between the depression and euthymia groups (*p* < 0.001); †comparison between the depression and euthymia groups (*p* < 0.01); §comparison between the mania and euthymia groups (*p* < 0.01); #comparison between the mania and euthymia groups (*p* < 0.05).

Figure 1A shows the IES-R results for stressful life events among the 3 groups. There were significant differences in the total IES-R scores (α = 0.92) observed among the 3 groups (*F* = 8.40, *p* = 0.001, power = 0.96). Post-hoc analysis showed significant differences between the groups; the IES-R total scores for the depression group (mean = 38.06, standard deviation [SD] = 16.56) and those for the mania group (mean 44.56, SD = 24.14) were significantly higher than those in the euthymia group (mean = 19.81, *SD* = 12.86; 95% confidence interval [CI] 7.57–28.94, *p* = 0.0005; 95% CI 7.62–41.88, *p* = 0.004, respectively). There were no significant differences in the total IES-R scores between the depression and the mania groups.

As shown in Figure 1A, in terms of the IES-R score sub-categories (intrusion, α = 0.89; avoidance, α = 0.84; and hyperarousal, α = 0.82), there were significant differences among the 3 groups for intrusion (*F* = 6.01, *p* = 0.004, power = 0.88) and hyperarousal (*F* = 9.42, *p* = 0.004, power = 0.98). For avoidance, the IES-R scores were significantly different among the 3 groups; however, the power did not reach 0.80 (*F* = 4.54, *p* = 0.015, power = 0.77). Each sub-category score, except for avoidance, was significantly higher in the depression and mania groups than in the euthymia group. For intrusion, the mean scores were as follows: depression group, 14.10 (*SD* = 7.37); mania group, 16.94 (*SD* = 9.89); and euthymia group, 7.56 (*SD* = 6.73). For avoidance, the mean scores were as follows: depression group, 14.74 (*SD* = 7.07); euthymia group, 8.25 (*SD* = 6.11); and mania group, 15.25 (*SD* = 9.88). For hyperarousal, the mean score of the depression group was 9.35 (*SD* = 4.99), and that of the mania group was 12.38 (*SD* = 7.99), while it was lower for the euthymia group (mean = 4.00, *SD* = 3.16).

#### IES-R scores for onset-related events

Figure 1B shows the IES-R scores for onset-related events among the 3 groups. There was a significant difference among the 3 groups in terms of the total IES-R scores (α = 0.94) as well as stressful life events scores (*F* = 10.59, *p* = 0.0001, power = 0.99). Post-hoc analysis showed significant differences between the groups: the total IES-R scores in the depression group (mean = 32.07, *SD* = 18.03) and those in the mania group (mean = 35.85, *SD* = 24.67) were significantly higher than those in the euthymia group (mean = 12.17, *SD* = 12.30; 95% CI 9.70–30.09, *p* = 0.0001; 95% CI 8.60–38.76, *p* = 0.002, respectively). There was no significant difference in the total scores between the depression and mania groups.

As shown in Figure 1B, for each sub-category of the IES-R score (intrusion, α = 0.90; avoidance, α = 0.88; and hyperarousal, α = 0.87), there were significant differences among the 3 groups: intrusion (*F* = 6.97, *p* = 0.002, power = 0.93), avoidance (*F* = 8.01, *p* = 0.001, power = 0.96), and hyperarousal (*F* = 9.53, *p* = 0.0002, power = 0.98). Each sub-category score of the depression and mania groups was significantly higher than that of the euthymia group. For intrusion, the mean score was as follows: depression group, 10.24 (*SD* = 7.38); mania group, 12.85 (*SD* = 9.86); euthymia group, 4.48 (*SD* = 5.38). For avoidance, the mean score of the depression group was 13.14 (*SD* = 7.53) and that of the mania group was 13.50 (*SD* = 10.25), while it was lower for euthymia (mean = 5.13, *SD* = 6.43). For hyperarousal, the mean score was as follows: depression group, 8.69 (*SD* = 5.39) and mania group, 9.50 (*SD* = 8.59), while it was lower for the euthymia group (mean = 2.57, *SD* = 2.79).

### Correlations of the IES-R score with the HDRS and YMRS scores

**Figure 2:**
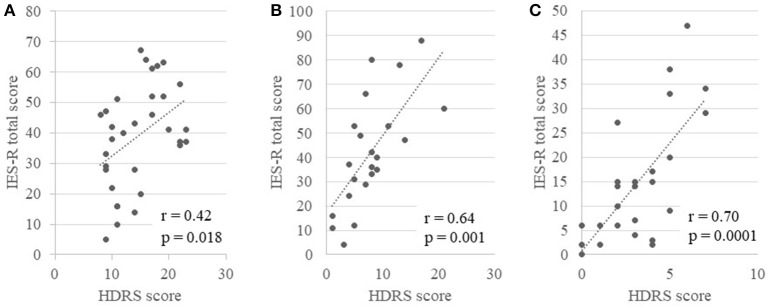
The correlation between the total score of the IES-R for life events related to PDSs or onset-related events and the HDRS for the 3 groups (**A**, depression group; **B**, mania group; and **C**, euthymia group).

Figures 2A–C show the correlations between the total IES-R scores for stressful life events or onset-related events and the HDRS scores for the 3 groups. Although some patients experienced both stressful life events and onset-related events, we adopted the higher IES-R score for each patient. There were significant positive correlations between the total IES-R score and the HDRS score for each group (depression group: *r* = 0.42, *p* = 0.018; mania group: *r* = 0.64, *p* = 0.001; euthymia group: *r* = 0.70, *p* = 0.0001). In terms of the intrusion score of the IES-R, there were significant positive correlations between the IES-R scores and the HDRS scores for each group (depression group: *r* = 0.43, *p* = 0.013; mania group: *r* = 0.63, *p* = 0.002; euthymia group: *r* = 0.55, *p* = 0.005). For avoidance, there were significant positive correlations between the IES-R scores and the HDRS scores in the mania and euthymia groups (mania group: *r* = 0.43, *p* = 0.044; euthymia group: *r* = 0.62, *p* = 0.001), while there was no significant correlation for the depression group. For hyperarousal, there were significant positive correlations between the IES-R scores and the HDRS scores for each group (depression group: *r* = 0.47, *p* = 0.006; mania group: *r* = 0.69, *p* = 0.0004; euthymia group: *r* = 0.61, *p* = 0.001).

There were significant positive correlations between the total IES-R scores and YMRS scores (*r* = 0.40, *p* = 0.05), and the hyperarousal score of the IES-R and YMRS (*r* = 0.41, *p* = 0.04) in the euthymia group, while no other significant correlations were observed in the other groups.

## Discussion

This study yielded two important findings. Firstly, even though stressful life events occur more frequently, and are less serious than traumatic experiences that meet the diagnostic criteria for PTSD, bipolar patients with a manic/hypomanic episode or a depressive episode perceived their experience of such stressful life events, including their onset-related events, as more severe PDSs than those in a euthymic state. Secondly, the severity of depressive symptoms, but not of manic symptoms, was positively correlated with the severity of the PDSs in patients with bipolar disorder, regardless of their mood episodes or euthymic state.

The first finding supports our hypothesis that patients with bipolar disorder experience PDSs associated with stressful life events similar to patients with unipolar major depression (8). Interestingly, this study shows that bipolar patients with a current manic or hypomanic episode also perceive their past stressful events as severe PDSs, similar to patients undergoing a current depressive episode. This study also demonstrates that patients with bipolar disorder in a euthymic state suffer less than those with any mood episode. In terms of depressive episodes, this finding supports those of our previous study, indicating that patients with treatment-refractory or unremitted unipolar major depression perceive their onset-related life events as serious PDSs (8). The finding that manic or hypomanic patients with bipolar disorder also experience PDSs associated with stressful past events was unexpected, as mood in a manic episode is often described as euphoric, cheerful, and high (23). However, considering that labile mood, which includes elevation, expansiveness, or irritability, is a clinical feature of bipolar mania (23), any mood episode may cause PDSs associated with stressful past events in patients with bipolar disorder. In addition, in terms of the psychoanalytic perspective, the hypothesis of manic defense that has been described by Klein (24), may help understand severe PDSs in bipolar patients with manic or hypomanic episode. When patients with bipolar disorder encounter stressful life events, they might exhibit manic symptoms as a defense against depression.

Our results demonstrated that the severity of depressive symptoms, but not that of manic symptoms, correlated positively with the severity of the PDSs in bipolar patients with any mood episodes and a euthymic state. According to the findings of this and our previous study (8), these results indicate that the PDSs associated with stressful past events may be related to depression in mood disorders, such as bipolar disorder and major depression. Experimental studies on human psychology, regarding the relationship between emotion and memory, provide clues to understand the association between depression and PDSs. Bower has advocated his network theory of affect which states that memories of emotional events are stored, connecting the places or situations related to them (25, 26). Therefore, people who feel certain emotions, such as happy or sad ones, are likely to recall the past events during which they had experienced the same emotions. This phenomenon is called mood-state-dependent memory (25, 26). Further, in terms of neural mechanisms of retrieval of memories, Anderson and colleagues, using functional magnetic resonance imaging (fMRI) studies, have reported that the suppression of intrusive or unwanted memories is regulated by inhibitory control through the connectivity between frontal cortices, involved in the prefrontal cortex and both the hippocampus and amygdala (27, 28). Considering these neural network theories, bipolar and unipolar depression patients with depressive symptoms might be predisposed to recall and ruminate past stressful events associated with a negative or sad emotion, and to perceive them as the PDSs.

To further investigate the association between depression and PDSs in patients with bipolar and unipolar disorders, experimental studies on biased autobiographical memory may contribute to understanding of the present findings. Such studies show an association of stressful past events with mood disorders, and accumulating evidence points toward negative recall bias in autobiographical memory. Autobiographical memory is thought to be the memory concerned with the recollection of personally experienced past events (29). It is thought that patients with depression recall and ruminate their past life events as negatively biased memories, based on impaired cognitive processing, and that these cognitive distortions and dysfunctions maintain the depressive mood (30, 31). Young et al. have reported abnormal activity of the amygdala and its related network, based on fMRI (32), and have demonstrated the effectiveness of real-time fMRI amygdala neurofeedback against major depression, based on the theory of biased autobiographical memory in patients with depression (33). As bipolar patients with any mood episodes also perceive stressful life events as PDSs, such as intrusive memories, future studies should investigate the association between bipolar disorder and biased autobiographical memory, in order to understand the pathophysiology of bipolar disorder.

Further, the severity of manic symptoms positively correlated with the severity of the PDSs associated with stressful life events in bipolar patients with euthymia, but not in those with manic or hypomanic or depressive episodes. It is difficult to interpret this finding, because the severities of manic symptoms and of PDSs associated with stressful events in euthymic patients with bipolar disorder are markedly less severe than those of patients with a manic or hypomanic episode. To address this issue, further detailed questionnaires or structural interviews regarding subthreshold manic symptoms should be conducted for euthymic patients with bipolar disorder.

This study has some limitations. Firstly, this study could not assess whether PDSs become more intense because of severe mood states or whether mood states were more severe because of more intense PDSs, due to its cross-sectional design. Prospective cohort studies are required to investigate this issue. In addition, this study could not exclude confounding factors. Secondly, the application of IES-R to assess PDSs in patients with bipolar disorder remains methodologically limited. Although PDSs associated with stressful life events were sufficiently covered by the items of the IES-R, the IES-R was originally developed as a tool for rating severity of PTSD. Further studies are required to develop and validate a reliable original tool for the assessment of PDSs. Thirdly, this study has been influenced by recall bias, because each participant was requested to recall past stressful events.

In conclusion, this study demonstrates that bipolar patients with a manic or hypomanic episode or a depressive episode perceive their experiences of stressful life events as more severe PDSs than do euthymic patients. Moreover, the severity of depressive symptoms, but not that of manic symptoms, positively correlates with the severity of the PDSs in bipolar patients with any mood episode and those in a euthymic state. These findings indicate that depression may closely correlate with PDSs associated with stressful past events in bipolar disorder.

## Author contributions

AS, TH, AK, and MI designed this study. AS and TH acquired data. AS, TH, and TN analyzed the data, and AS, TH, TN, and MI interpreted the results. AS and TH drafted the manuscript, and AK, TN, and MI revised the manuscript. MI supervised the study. All authors approved the final manuscript.

### Conflict of interest statement

The authors declare that the research was conducted in the absence of any commercial or financial relationships that could be construed as a potential conflict of interest. The reviewer SS and handling Editor declared their shared affiliation.

## Abbreviations

DSM: Diagnostic and Statistical Manual of Mental Disorders
HDRS: Hamilton Depression Rating Scale
IES-R: Impact of Event Scale-Revised
PDSs: psychological distress symptoms
PTSD: posttraumatic stress disorder
YMRS: Young Mania Rating Scale.

## References

[1] Hammen C Gitlin M. Stress reactivity in bipolar patients and its relation to prior history of disorder. Am J Psychiatry (1997) 154:856–57. 10.1176/ajp.154.6.856 9167516

[2] Horesh N Iancu I A comparison of life events in patients with unipolar disorder or bipolar disorder and controls. Compr Psychiatry (2010) 51:157–64. 10.1016/j.comppsych.2009.05.005 20152296

[3] Johnson SL Miller I. Negative life events and time to recovery from episodes of bipolar disorder. J. Abnormal Psychol. (1997) 106:449–57. 10.1037/0021-843X.106.3.449 9241946

[4] Johnson L Andersson-Lundman G Aberg-Wistedt A Mathe AA. Age of onset in affective disorder: its correlation with hereditary and psychosocial factors. J Affect Disord. (2000) 59:139–48. 10.1016/S0165-0327(99)00146-9 10837882

[5] Hosang GM Korszun A Jones L Jones I Gray JM Gunasinghe CM. Adverse life event reporting and worst illness episodes in unipolar and bipolar affective disorders: measuring environmental risk for genetic research. Psychol Med. (2010) 40:1829–37. 10.1017/S003329170999225X 20132580

[6] Meiser-Stedman R Dalgleish T Yule W Smith P. Intrusive memories and depression following recent non-traumatic negative life events in adolescents. J Affect Disord. (2012) 137:70–8. 10.1016/j.jad.2011.12.020 22244376

[7] Mol SS Arntz A Metsemakers JF Dinant GJ Vilters-van Montfort PA Knottnerus JA. Symptoms of post-traumatic stress disorder after non-traumatic events: evidence from an open population study. Br. J. Psychiatry (2005) 186:494–99. 10.1192/bjp.186.6.494 15928360

[8] Kimura A Hashimoto T Niitsu T Iyo M. Presence of psychological distress symptoms associated with onset-related life events in patients with treatment-refractory depression. J Affect Disord. (2015) 175:303–9. 10.1016/j.jad.2015.01.027 25661396

[9] Ghaemi SN Ko JY Goodwin FK. “Cade's disease” and beyond: misdiagnosis, antidepressant use, and a proposed definition for bipolar spectrum disorder. Can J Psychiatry (2002) 47:125–34. 10.1177/070674370204700202 11926074

[10] Angst J Gamma A Benazzi F Ajdacic V Eich D Rossler W. Diagnostic issues in bipolar disorder. Eur Neuropsychopharmacol. (2003) 13(Suppl. 2):S43–50. 10.1016/S0924-977X(03)00077-4 12957719

[11] Angst J Gamma A Benazzi F Ajdacic V Eich D Rossler W. Toward a re-definition of subthreshold bipolarity: epidemiology and proposed criteria for bipolar-II, minor bipolar disorders and hypomania. J Affect Disord. (2003) 73:133–46. 10.1016/S0165-0327(02)00322-1 12507746

[12] American Psychiatric Association Diagnostic and Statistical Manual of Mental Disorders, 4th Edn. Washington, DC: American Psychiatric Publishing (2013).

[13] Otsubo T Tanaka K Koda R Shinoda J Sano N Tanaka S. Reliability and validity of Japanese version of the Mini-International Neuropsychiatric Interview. Psychiatry Clin Neurosci. (2005) 59:517–26. 10.1111/j.1440-1819.2005.01408.x 16194252

[14] Sheehan DV Lecrubier Y Sheehan KH Amorim P Janavs J Weiller E. The Mini-International Neuropsychiatric Interview (M.I.N.I.): the development and validation of a structured diagnostic psychiatric interview for DSM-IV and ICD-10. J Clin Psychiatry (1998) 59(Suppl. 20): 22–33; quiz 34–57. 9881538

[15] Hamilton M. Development of a rating scale for primary depressive illness. Br J Soc Clin Psychol. (1967) 6:278–96. 10.1111/j.2044-8260.1967.tb00530.x 6080235

[16] Williams JB A structured interview guide for the Hamilton Depression Rating Scale. Archives of general psychiatry (1988) 45:742–7. 10.1001/archpsyc.1988.01800320058007 3395203

[17] Young RC Biggs JT Ziegler VE Meyer DA. A rating scale for mania: reliability, validity and sensitivity. Br J Psychiatry (1978) 133:429–35. 10.1192/bjp.133.5.429 728692

[18] First MB Structured Clinical Interview for DSM-IV Axis I Disorders: SCID - I: Clinician Version: Administration Booklet. Washington, DC: American Psychiatric Press (1997).

[19] Brugha TS Cragg D. The List of Threatening Experiences: the reliability and validity of a brief life events questionnaire. Acta Psychiatr Scand. (1990) 82:77–81. 10.1111/j.1600-0447.1990.tb01360.x 2399824

[20] Weiss DS Marmar CR The Impact of Event Scale-Revised. New York, NY: Guilford Press (1997).

[21] Baumert J Simon H Gundel H Schmitt C Ladwig KH. The Impact of Event Scale–Revised: evaluation of the subscales and correlations to psychophysiological startle response patterns in survivors of a life-threatening cardiac event: an analysis of 129 patients with an implanted cardioverter defibrillator. J Affect Disord. (2004) 82:29–41. 10.1016/j.jad.2003.09.006 15465574

[22] Asukai N Kato H Kawamura N Kim Y Yamamoto K Kishimoto J Reliability and validity of the Japanese-language version of the impact of event scale-revised (IES-R-J): 4 studies of different traumatic events. J. Nerv. Ment. Dis. (2002) 190:175–82. 10.1097/00005053-200203000-00006 11923652

[23] Cowen P Harrison P Burns T Shorter Oxford Textbook of Psychiatry, 6th Edn. Oxford University Press (2012). p. 210–2.

[24] Klein M A contribution to the psychogenesis of manic-depressive states. Int J Psychoanal. (1935) 16:145–74.

[25] Bower GH. Mood and memory. American Psychologist (1981) 36:129–48. 10.1037/0003-066X.36.2.129 7224324

[26] Bower GH How might emotions affect learning? In: Christianson S-Å editor. The Handbook of Emotion and Memory: Research and Theory. Hillsdale, NJ: Lawrence Erlbaum Associates (1992). p. 3–31.

[27] Anderson MC Ochsner KN Kuhl B Cooper J Robertson E Gabrieli SW. Neural systems underlying the suppression of unwanted memories. Science (2004) 303:232–5. 10.1126/science.1089504 14716015

[28] Gagnepain P Hulbert J Anderson MC. Parallel regulation of memory and emotion supports the suppression of intrusive memories. J Neurosci. (2017) 37:6423–41. 10.1523/JNEUROSCI.2732-16.2017 28559378 PMC5511877

[29] Williams JM Barnhofer T Crane C Herman D Raes F Watkins E. Autobiographical memory specificity and emotional disorder. Psychol Bull. (2007) 133:122–48. 10.1037/0033-2909.133.1.122 17201573 PMC2834574

[30] Gotlib IH Joormann J. Cognition and depression: current status and future directions. Annu Rev Clin Psychol. (2010) 6:285–312. 10.1146/annurev.clinpsy.121208.131305 20192795 PMC2845726

[31] Ahmadpanah M Astinsadaf S Akhondi A Haghighi M Sadeghi Bahmani D Nazaribadie M. Early maladaptive schemas of emotional deprivation, social isolation, shame and abandonment are related to a history of suicide attempts among patients with major depressive disorders. Compr Psychiatry (2017) 77:71–9. 10.1016/j.comppsych.2017.05.008 28636896

[32] Young KD Siegle GJ Bodurka J Drevets WC. Amygdala activity during autobiographical memory recall in depressed and vulnerable individuals: association with symptom severity and autobiographical overgenerality. Am J Psychiatry (2016) 173:78–89. 10.1176/appi.ajp.2015.15010119 26541813

[33] Young KD Siegle GJ Zotev V Phillips R Misaki M Yuan H. Randomized clinical trial of real-time fMRI amygdala neurofeedback for major depressive disorder: effects on symptoms and autobiographical memory recall. Am J Psychiatry (2017) 174:748–55. 10.1176/appi.ajp.2017.16060637 28407727 PMC5538952

